# Clinical phenotypes and genetic analysis of 30 children with Gitelman syndrome

**DOI:** 10.3389/fped.2026.1853435

**Published:** 2026-06-23

**Authors:** Wenyan Wang, Fei Zhao, Guixia Ding, Songming Huang, Xueqin Cheng

**Affiliations:** Department of Nephrology, Children’s Hospital of Nanjing Medical University, Nanjing, Jiangsu, China

**Keywords:** children, clinical phenotype, genetic, Gitelman syndrome, SLC12A3

## Abstract

**Objective:**

To investigate the clinical phenotype, SLC12A3 gene spectrum, and genotype–phenotype correlation in Chinese children with Gitelman syndrome (GS).

**Methods:**

Clinical and genetic data of 30 genetically confirmed pediatric GS cases (2015–2025) were retrospectively analyzed. Patients were grouped by sex, serum potassium level, and mutation functional domain.

**Results:**

Mean onset age was 7.9 ± 3.4years. Common manifestations included muscle weakness (50%) and limb numbness (40%). All patients had hypokalemia; 90% had hypomagnesemia. Female patients exhibited more limb numbness and lower serum calcium. Severe hypokalemia (<2.5 mmol/L) was associated with tetany, dyslipidemia, and increased urinary potassium excretion. Genetic testing identified 55 SLC12A3 variants (6 novel), with high-frequency mutations c.1456G > A (p.D486N) and c.179C > T (p.T60M). No phenotypic difference was found based on functional domain classification.

**Conclusion:**

Pediatric GS shows significant clinical heterogeneity. Severe hypokalemia is accompanied by early dyslipidemiaindicates. SLC12A3 variants are diverse; functional domain alone cannot predict phenotype. Early screening and long-term follow-up are essential for optimal management.

## 1 Introduction

Gitelman syndrome (GS) is an autosomal recessive salt-losing tubulopathy that was first described in 1966 (1, 2). The causative gene has been identified as *SLC12A3*, which encodes the thiazide-sensitive sodium-chloride cotransporter (NCC) located in the distal convoluted tubule (DCT) of the kidney (3–5). The biochemical hallmarks of GS include hypokalemia, metabolic alkalosis, hypomagnesemia, hypocalciuria, and secondary activation of the renin-angiotensin-aldosterone system (RAAS), affected patients typically present with normotension or hypotension (1, 6–8). Currently, GS is recognized as the most prevalent hereditary renal tubular disease worldwide, with an estimated incidence ranging from 1 to 10 cases per 40,000 individuals. Notably, its prevalence varies substantially across ethnic groups. East Asian populations exhibit a higher disease burden, and the carrier-derived prevalence in Japan reaches up to 10.3 per 10,000 people (1, 6, 9).

The clinical manifestations of GS are highly heterogeneous. Reported manifestations range from asymptomatic (especially in children, with over 30% having no obvious symptoms) and non-specific symptoms (such as fatigue, salt craving, polyuria) to severe neuromuscular symptoms (such as muscle weakness, cramps, and tetany) (9–12). Diagnosis is often delayed until adolescence or adulthood (9). This clinical heterogeneity not only poses diagnostic challenges but may also lead to unrecognized and poorly managed long-term complications. Studies have confirmed that chronic electrolyte disturbances such as hypokalemia and hypomagnesemia are closely associated with growth retardation in children with GS and chondrocalcinosis in adulthood, and can significantly reduce patients' quality of life (6, 11, 13–15). In addition, adult GS patients often have abnormal glucose metabolism (including impaired glucose tolerance and diabetes mellitus), indicating that GS is not a single renal disease but a multisystem metabolic disorder (13, 16).

Genetically, biallelic loss-of-function mutations in *SLC12A3* are the definitive pathogenic cause of GS. To date, more than 500 mutations in *SLC12A3* have been identified, with missense mutations being the most common (6, 17, 18). For the Chinese GS population, p.Thr60Met (T60M) and p.Asp486Asn (D486N) have been consistently identified as two prevalent hotspot mutations across multiple independent cohorts (17, 19, 20). Elucidating the association between genotype and clinical phenotype (genotype–phenotype correlation) is crucial for understanding disease heterogeneity, predicting disease progression, and identifying potential complications. Nevertheless, existing relevant conclusions remain controversial. For instance, no consensus has been reached regarding whether biallelic homozygous variants correlate with aggravated electrolyte disorders or whether certain specific mutations are correlated with extra-renal complications (17, 21–23). These conflicting findings suggest that GS phenotypic variability is modulated by multiple confounding factors, including mutation classification, modifier genes, and external environmental cues.

Given the aforementioned challenges, including heterogeneous clinical phenotypes, frequent diagnostic delays, irreversible long-term metabolic complications, and elusive genotype-phenotype associations, targeted analyses focusing on the Chinese pediatric GS cohort are of great clinical significance and research value. Population-specific data can help delineate the real-world clinical and genetic landscape of GS, summarize age-specific phenotypic characteristics, validate established high-frequency mutations, and provide novel clues for unresolved genotype-phenotype relationships. Accordingly, the present retrospective study enrolled 30 genetically confirmed pediatric GS patients treated in our medical center from 2015 to 2025. We aimed to comprehensively characterize their clinical manifestations, laboratory profiles, and SLC12A3 mutational spectrum, and further explore potential genotype-phenotype correlations. Our findings are expected to optimize early recognition, precision diagnosis, and individualized long-term management strategies for childhood GS.

## 2 Methods and materials

### 2.1 Study population

This retrospective observational study enrolled pediatric patients with genetically confirmed Gitelman syndrome (GS) admitted to the Children's Hospital of Nanjing Medical University from January 1, 2015, to January 1, 2025. All participants were screened according to unified inclusion and exclusion criteria formulated based on the Chinese Expert Consensus on the Diagnosis and Management of Gitelman Syndrome (2021 Edition) (11). The inclusion criteria were as follows: (1) clinical symptoms and biochemical indicators consistent with the diagnostic criteria for pediatric GS; (2) genetic testing identified pathogenic or likely pathogenic variants in the SLC12A3 gene. Patients without any detectable SLC12A3 gene variants via genetic analysis were excluded from this study. The enrolled patients were stratified into different subgroups for stratified analysis. Grouping strategies included gender stratification (male and female subgroups) and serum potassium stratification. According to the hypokalemia grading standard proposed by the above expert consensus, patients were divided into a mild-to-moderate hypokalemia group (serum potassium ≥ 2.5 mmol/L) and a severe hypokalemia group (serum potassium < 2.5 mmol/L) (11). This study was approved by the Medical Ethics Committee of the Children's Hospital of Nanjing Medical University (Ethics Approval No: 202603041-1), and all relevant clinical data were used for research purposes only.

### 2.2 Data collection

All clinical data analyzed in this study were retrospectively extracted from the hospital's standardized electronic medical record system by two independent researchers to reduce selection and recording bias. The collected data covered baseline demographic information, clinical phenotypic characteristics and complete laboratory biochemical indicators obtained during the initial hospitalization of patients. Baseline demographic data consisted of patient sex and age at initial symptom onset. Collected clinical phenotypes included common GS-related manifestations, such as short stature, muscle weakness, limb numbness, fatigue and other associated symptoms. For laboratory parameters, all blood samples were collected after overnight fasting and detected by the hospital's certified clinical laboratory department with unified testing reagents and instruments. The retrieved indicators included the lowest levels of serum potassium and magnesium recorded during the first hospitalization, as well as serum sodium, chloride, calcium, blood lipid profiles (triglyceride, high-density lipoprotein and total cholesterol), renal function indexes (serum creatinine and urea nitrogen), renin–angiotensin–aldosterone system indicators (plasma renin activity, angiotensin I, angiotensin II and aldosterone), and 24-hour urinary electrolyte excretion levels.

### 2.3 Genetic testing

After obtaining informed consent from the guardians, 2 mL of peripheral venous blood was collected from each child and their parents. Genomic DNA was extracted using a Blood Genomic DNA Extraction Kit (Tiangen Biotech Co., Ltd., Beijing) and sent to MyGenostics (Beijing) for whole-exome sequencing (WES) on the Illumina HiSeq high-throughput sequencer. Allele frequencies were queried for all loci. WES results were referenced to the gnomAD database, and variants with a frequency <0.01 were filtered out. We further searched the HGMD database and relevant literature, and combined the proband's clinical data with prediction results from bioinformatics software (PolyPhen-2, MutationTaster, GERP++). Variants were classified according to the American College of Medical Genetics and Genomics (ACMG) variant classification criteria. All WES-identified variants in the children and their parents were verified by Sanger sequencing.

### 2.4 Statistical analysis

All statistical analyses were performed using the Statistical Package for the Social Sciences (SPSS) software, version 19.0. Continuous variables were expressed as the mean ± standard deviation (*x¯* ± *s*) if they followed a normal distribution, and group comparisons were performed using the independent-samples *t*-test. If the data were not normally distributed, they were expressed as the median (interquartile range) [*M* (Q1, Q3)] and analyzed using the Mann–Whitney *U* test (rank-sum test). Categorical variables were presented as the number of cases (percentage) [*n* (%)]. Between-group comparisons for categorical data were conducted using the chi-square test or, when the expected frequency in any cell was less than 5, Fisher's exact test was used. A two-tailed *P*-value <0.05 was considered statistically significant for all tests.

## 3 Results

### 3.1 Clinical analysis

#### 3.1.1 General characteristics and clinical data

Of the 30 enrolled children diagnosed with GS, 23 (76.7%) were male and 7 (23.3%) were female. The age at symptom onset ranged from 1.25 to 15.17 years, with a mean value of 7.9 ± 3.4 years. The most prevalent clinical manifestation was muscle weakness (15 cases, 50.0%), followed by limb numbness (12 cases, 40.0%) and short stature (11 cases, 36.7%). Other observed symptoms included vomiting (9 cases, 30.0%), fever (7 cases, 23.3%), polydipsia and polyuria (4 cases, 13.3%), tetany, constipation, and abdominal pain (3 cases each, 10.0%), salt craving (2 cases, 6.7%), and dehydration (1 case, 3.3%). All patients presented with hypokalemia, with the mean minimum serum potassium level of 2.5 ± 0.3 mmol/L; 90% of the cohort exhibited concomitant hypomagnesemia at initial presentation. No obvious abnormalities were detected in other serum electrolytes, renal function indicators or hemoglobin levels. Mild dyslipidemia was identified in partial patients, with average triglyceride, high-density lipoprotein and total cholesterol levels of 1.2 ± 0.7, 1.4 ± 0.4 and 4.5 ± 0.9 mmol/L, respectively. RAAS profiling was performed in 28 patients, among whom 75.0% showed renin hyperactivation. Additionally, all enrolled children displayed varying degrees of elevated 24-hour urinary potassium excretion (Table 1).

**Table 1:** Clinical characteristics and sex differences in pediatric patients with Gitelman syndrome.

Variable	Total (*n* = 30)	Male (*n* = 23)	Female (*n* = 7)	Statistic (*z*/*t*)	*P*-value
General characteristics					
Age at onset (years)	7.9 ± 3.4	7.8 ± 3.5	8.6 ± 3.1	−0.6	0.6
Height (SD)	−1.1 ± 1.3	−1.1 ± 1.2	−1.2 ± 1.5	0.3	0.8
Clinical features, *n* (%)					
Short stature	11 (36.7)	8 (34.8)	3 (42.9)		1
Muscle weakness	15 (50)	9 (39.1)	6 (85.7)		0.1
Limb numbness	12 (40)	6 (26.1)	6 (85.7)*		0.0
Tetany	3 (10)	1 (4.3)	2 (28.6)		0.1
Polydipsia and polyuria	4 (13.3)	2 (8.7)	2 (28.6)		0.2
Vomiting	9 (30)	8 (34.8)	1 (14.3)		0.4
Abdominal pain and distension	3 (3)	3 (13)	0 (0)		1
Constipation	3 (10)	3 (13)	0 (0)		1
Fever	7 (23.3)	4 (17.4)	3 (42.9)		0.3
Salt craving	2 (6.7)	1 (4.3)	1 (14.3)		0.4
Dehydration	1 (3.3)	1 (4.3)	0 (0)		1
Laboratory examinations					
pH	7.4 ± 0.1	7.4 ± 0.1	7.5 ± 0.1	−1.8	0.1
Hypokalemia	30 (100)	23 (100)	7 (100)		
Lowest serum K^+^ (mmol/L)	2.5 ± 0.3	2.6 ± 0.3	2.3 ± 0.4	1.9	0.1
Hypomagnesemia	27 (90)	22 (95.6)	5 (71.4)		
Serum Mg^2+^ (mmol/L)	0.7 ± 0.1	0.7 ± 0.1	0.7 ± 0.1	−1.5	0.1
Serum Na^+^ (mmol/L)	137.6 (135.7, 139.4)	137.8 (135.7, 140)	139 (135.4, 139.1)	−0.1	0.9
Serum Cl^−^ (mmol/L)	96.1 (94.2, 97.9)	97 (94, 98.2)	97.3 (94.8, 97.7)	−0.2	0.9
Serum Ca^2+^ (mmol/L)	2.5 ± 0.1	2.5 ± 0.1	2.4 ± 0.2*	2.1	0.0
Triglyceride (mmol/L)	1.2 ± 0.7	0.9 ± 0.3	1.7 ± 1.1	−1.8	0.1
HDL-C (mmol/L)	1.4 ± 0.4	1.5 ± 0.4	1.0 ± 0.1*	5.1	0.0
Total cholesterol (mmol/L)	4.5 ± 0.9	4.6 ± 0.8	4.2 ± 1.1	1.1	0.3
BUN (mmol/L)	4.3 ± 1.5	4.4 ± 1.6	4.2 ± 1.5	0.3	0.8
Scr (umol/L)	34.5 ± 7.7	33.7 ± 7.9	36.9 ± 6.9	−0.9	0.3
Uric acid (umol/L)	247.9 ± 102.2	252.6 ± 110.7	232.9 ± 72.3	0.4	0.7
Hemoglobin (g/L)	135 (130, 141)	133 (131, 139)	143 (130, 144)	−0.6	0.6
RASS (Supine)	28	21	7		
PRA [ng/(mL·h)]	5.4 (1.9, 9.6)	4.5 (1.7, 9.1)	6.2 (2.9, 10.5)	−0.9	0.3
Angiotensin I (ug/L)	2.3 (1.4, 3.8)	1.9 (1.1, 3.1)	3.3 (2.2, 7.3)	−1.8	0.1
Angiotensin II (ng/L)	84.6 (67.2, 94.7)	81.8 (67.4, 92.9)	89.6 (61.8, 103.8)	−1.0	0.3
Aldosterone (ng/L)	167.2 (129.9, 262.4)	147.3 (124.8, 202.0)	208.2 (142.5, 265.5)	−1.2	0.2
24 h Urinary electrolytes	27	20	7		
Urinary K^+^ [mmol/(kg·day)]	1.8 (1.3, 2.6)	1.7 (1.2, 2.5)	2.1 (1.8, 2.9)	−1.6	0.1
Urinary Mg^2+^ [mmol/(kg·day)]	0.1 ± 0.1	0.1 ± 0.1	0.1 ± 0.0	0.8	0.4
Urinary Na^+^ [mmol/(kg·day)]	5.3 ± 2.7	5.8 ± 3.0	4 ± 1.2	1.5	0.1
Urinary Cl^−^ [mmol/(kg·day)]	5.6 ± 2.9	6.0 ± 3.3	4.7 ± 0.9	1.0	0.3
Urinary Ca^2+^ [mmol/(kg·day)]	0.006 (0.003, 0.014)	0.006 (0.004, 0.008)	0.011 (0.001, 0.025)	−0.0	0.9
Urinary P [mmol/(kg·day)]	0.4 (0.3, 0.5)	0.4 (0.3, 0.6)	0.3 (0.2, 0.4)	−1.0	0.3

SD, standard deviation; HDL-C, high-density lipoprotein cholesterol; BUN, blood urea nitrogen; Scr, serum creatinine; RAAS, renin–angiotensin–aldosterone system. This table summarizes the demographic, clinical symptoms, and laboratory parameters of 30 children diagnosed with Gitelman syndrome and compares relevant indicators between male and female subgroups. Baseline information, predominant clinical manifestations, serum biochemical indices, RAAS activity, and 24-hour urinary electrolytes are comprehensively listed in the table. Hypokalemia was identified in all enrolled patients, and the majority of children presented with concomitant hypomagnesemia. Females exhibited significantly higher limb numbness, lower serum calcium, and lower HDL-C levels compared with males. This table indicates obvious phenotypic heterogeneity and distinct gender-specific discrepancies among pediatric GS patients.

* *P* < 0.05 vs. the male group.

#### 3.1.2 Group analysis by sex

Subgroup analysis stratified by gender demonstrated no significant between-group differences in age at symptom onset and height. Regarding clinical phenotypes, female patients exhibited a markedly higher incidence of limb numbness compared with male counterparts (*P* < 0.05), whereas the remaining clinical manifestations were comparable across the two groups. In terms of biochemical indicators, females had lower serum calcium levels (2.4 ± 0.2 vs. 2.5 ± 0.1 mmol/L) and high-density lipoprotein concentrations (1.0 ± 0.1 vs. 1.5 ± 0.4 mmol/L), and both discrepancies reached statistical significance (both *P* < 0.05). No gender-based disparities were observed in RAAS-related parameters and 24-hour urinary electrolyte profiles (Table 1).

#### 3.1.3 Group analysis by serum potassium level

No significant differences in age at onset and height were observed between the mild-to-moderate and severe hypokalemia groups. Tetany exclusively occurred in patients with severe hypokalemia, presenting a remarkable intergroup difference, whereas the remaining clinical manifestations were comparable between the two cohorts. Regarding laboratory indicators, the severe hypokalemia group exhibited notably lower serum sodium levels (136.3 ± 2.6 vs. 138.3 ± 2.2 mmol/L) than the mild-to-moderate group (*P* < 0.05). Additionally, the severe group had elevated triglyceride levels [1.3 (0.9, 1.7) mmol/L] and decreased high-density lipoprotein levels (1.1 ± 0.3 mmol/L) compared with the mild-to-moderate group [0.9 (0.8, 1.2) mmol/L and 1.5 ± 0.4 mmol/L, respectively], and both differences reached statistical significance (both *P* < 0.05). Moreover, 24-hour urinary potassium excretion was markedly increased in the severe hypokalemia group (*P* < 0.05). No obvious disparities in other urinary electrolyte profiles and RAAS parameters were identified across subgroups (Table 2).

**Table 2:** Clinical and laboratory differences according to serum potassium level in pediatric patients with Gitelman syndrome.

Variable	The mild-to-moderate hypokalemia group (*n* = 19)	The severe hypokalemia group (*n* = 11)	Statistic (*z*/*t*)	*P*-value
General characteristics				
Age at onset (years)	7.6 ± 3.1	8.5 ± 3.8	−0.7	0.5
Height (SD)	−0.9 ± 1.3	−1.3 ± 1.2	0.8	0.4
Clinical features, *n* (%)				
Short stature	7 (36.8)	4 (36.3)		1
Muscle weakness	8 (42.1)	7 (63.6)		0.5
Limb numbness	5 (26.3)	7 (63.6)		0.1
Tetany	0 (0)	3 (27.3)*		0.0
Polydipsia and polyuria	2 (10.5)	2 (18.2)		0.6
Vomiting	5 (26.3)	4 (36.3)		0.7
Abdominal pain and distension	2 (10.5)	1 (9.1)		1
Constipation	2 (10.5)	1 (9.1)		1
Fever	3 (15.7)	4 (36.3)		0.4
Salt craving	2 (10.5)	0 (0)		0.5
Dehydration	0 (0)	1 (9.1)		0.4
Laboratory examinations				
pH	7.4 ± 0.0	7.5 ± 0.0	−1.8	0.1
Serum Mg^2+^ (mmol/L)	0.7 ± 0.1	0.7 ± 0.1	−0.6	0.6
Serum Na^+^ (mmol/L)	138.3 ± 2.2	136.3 ± 2.6*	2.3	0.0
Serum Cl^−^ (mmol/L)	97.2 (94.3, 98.3)	96.1 (93.1, 97.7)	−1.1	0.3
Serum Ca^2+^ (mmol/L)	2.5 ± 0.13	2.4 ± 0.147	0.7	0.5
Triglyceride (mmol/L)	0.9 (0.8, 1.2)	1.3 (0.9, 1.7)*	−2.2	0.0
HDL-C (mmol/L)	1.5 ± 0.4	1.1 ± 0.3*	2.7	0.0
Total cholesterol (mmol/L)	4.5 ± 0.8	4.3 ± 0.9	0.6	0.6
BUN (mmol/L)	4.7 ± 1.1	3.6 ± 1.9	1.9	0.1
Scr (umol/L)	33.8 ± 7.5	35.6 ± 8.3	−0.6	0.6
Uric acid (umol/L)	233 (196, 310)	198.6 (121, 319)	−1.1	0.3
Hemoglobin (g/L)	136.8 ± 8.1	134.5 ± 6.7	0.8	0.4
RASS (Supine)	17	11		
PRA [ng/(mL·h)]	4.6 (1.7.8.4)	6.2 (2.4, 10.2)	−0.6	0.6
Angiotensin I (ug/L)	1.9 (1.1, 2.9)	3.3 (1.9, 5.2)	−1.7	0.1
Angiotensin II (ng/L)	72.7 (63.3, 88.3)	91.6 (80.8, 103.8)	−1.4	0.2
Aldosterone (ng/L)	128.2 (180.7, 264.9)	140.4 (130.3, 207.4)	−0.9	0.4
24 h Urinary electrolytes	16	11		
Urinary K^+^ [mmol/(kg·day)]	1.6 ± 0.6	2.8 ± 1.3*	−2.8	0.0
Urinary Mg^2+^ [mmol/(kg·day)]	0.1 ± 0.1	0.1 ± 0.0	−0.3	0.8
Urinary Na^+^ [mmol/(kg·day)]	5.4 ± 2.6	5.2 ± 3.1	0.2	0.9
Urinary Cl^−^ [mmol/(kg·day)]	5.1 ± 2.3	6.4 ± 3.6	−1.2	0.3
Urinary Ca^2+^ [mmol/(kg·day)]	0.006 (0.003, 0.009)	0.007 (0.005.0.016)	−0.7	0.5
Urinary P [mmol/(kg·day)]	0.3 (0.3, 0.5)	0.416 (0.3, 0.7)	−1.2	0.2

SD, standard deviation; HDL-C, high-density lipoprotein cholesterol; BUN, blood urea nitrogen; Scr, serum creatinine; RAAS, renin–angiotensin–aldosterone system. This table summarizes the clinical manifestations and laboratory profiles of 30 children diagnosed with Gitelman syndrome and analyzes relevant indicators between mild-to-moderate and severe hypokalemia subgroups. The included variables cover predominant clinical symptoms, serum biochemical indices, RAAS activity, and 24-hour urinary electrolytes. Compared with patients in the mild-to-moderate group, individuals with severe hypokalemia presented a higher frequency of tetany, decreased serum sodium levels, elevated triglyceride concentrations, and increased 24-hour urinary potassium excretion. Collectively, these findings reveal a close correlation between hypokalemia severity and corresponding clinical and laboratory alterations among pediatric GS patients.

* *P* < 0.05 vs. the mild-to-moderate hypokalemia group.

### 3.2 Genetic analysis

**Figure 1:**
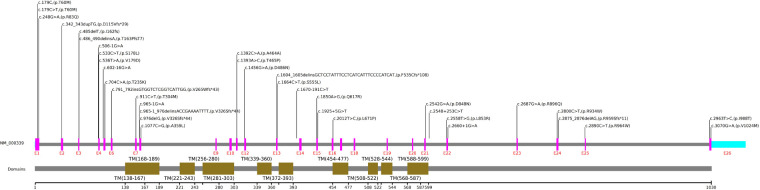
Schematic diagram of the SLC12A3 gene and protein structure. The upper panel depicts the genomic organization of the *SLC12A3* gene (NM_000339), including 26 pink-highlighted exons (E1–E26). All identified variants are annotated above relevant exons with corresponding cDNA and amino acid changes. The middle panel illustrates the structural domains of sodium-chloride cotransporter (NCC), covering multiple transmembrane domains (brown boxes) and a C-terminal intracellular domain (cyan box). The bottom panel shows the amino acid position scale. Overall, *SLC12A3* mutations scattered throughout the entire gene, and most variants concentrated in functionally vital transmembrane regions, indicating prominent genetic heterogeneity among children with Gitelman syndrome.

Only SLC12A3 variants were detected in all 30 children, with no CLCNKB variants found. There were 3 homozygotes (Cases 18, 20, 24), 24 compound heterozygotes, 1 child with two SLC12A3 variants both of maternal origin (Case 22), and 2 children carrying only one pathogenic variant (Cases 19, 28). Based on the NM_001126108 transcript, 55 distinct variants were identified in the 30 patients, including 32 missense variants (58.2%), 11 frameshift variants (20.0%), 5 splice-site variants (9.1%), 4 non-coding region variants (7.3%), 2 exon deletion variants (3.6%), and 1 synonymous variant (1.8%). Six novel unreported variants were identified: c.2531T > G(p.L844R) (PM2_Supporting + PM3 + PP3), c.704C > A(p.T235K), c.2800C > T(p.R934W) (PM2_Supporting + PM3 + PP3), c.1604_1605delinsGCTCCTATTTCCTCATCATTTCCCCATCAT(p.F535Cfs108) (PVS1 + PM1 + PM2_Supporting + PM3), c.1925 + 5G > T (PM2_Supporting + PM3_Supporting + PP3), and c.1392C > A (p.A464A) (PP3). Five high-frequency mutations were also detected: c.1456G > A (p.D486N) in 8 cases, c.965-1_976delinsACCGAAAATTTT (p.V326Sfs44) in 5 cases, c.179C > T (p.T60M) in 3 cases, c.533C > T (p.S178L) in 2 cases, and exon20-24 deletion in 2 cases (Figure 1).

### 3.3 Analysis of phenotype–genotype relationship

Figure 1 illustrates the schematic structure and protein functional domains of the *SLC12A3* gene. All enrolled patients were categorized into functional and non-functional domain mutation groups according to the positional distribution of pathogenic variants within protein functional regions. The functional domain mutation group was defined as biallelic variants exclusively located in functional domains. By contrast, patients carrying either a single functional-domain variant or biallelic variants confined to non-functional domains were assigned to the non-functional domain mutation group. Subgroup analysis indicated no significant differences in age at onset, height, clinical phenotypes, serum biochemical parameters, RAAS activity, and 24-hour urinary electrolyte levels between the two groups (Table 3).

**Table 3:** Clinical phenotype and genetic characteristics in pediatric patients with Gitelman syndrome.

Variable	The non-functional domain mutation group (*n* = 21)	The functional domain mutation group (*n* = 9)	Statistic (*z*/*t*)	*P*-value
General characteristics				
Age at onset (years)	7.9 ± 3.6	8.0 ± 2.9	−0.1	0.9
Height (SD)	−0.9 ± 1.3	−1.4 ± 1.3	0.8	0.4
Clinical features, *n* (%)				
Short stature	7 (33.3)	4 (44.4)		0.7
Muscle weakness	10 (47.6)	5 (55.6)		1
Limb numbness	9 (42.8)	3 (33.3)		0.7
Tetany	3 (14.3)	0 (0)		0.5
Polydipsia and polyuria	2 (9.5)	2 (22.2)		0.6
Vomiting	6 (28.6)	3 (33.3)		1
Abdominal pain and distension	1 (4.8)	2 (22.2)		0.2
Constipation	2 (9.5)	1 (11.1)		1
Fever	6 (28.5)	1 (11.1)		0.4
Salt craving	0 (0)	2 (22.2)		0.1
Dehydration	1 (4.8)	0 (0)		1
Laboratory examinations				
pH	7.4 ± 0.4	7.4 ± 0.1	1.3	0.2
Hypokalemia	21 (100)	9 (100)		
Lowest serum K^+^ (mmol/L)	2.6 (2.3, 2.8)	2.6 (2.5, 2.7)	−0.5	0.6
Hypomagnesemia	19 (90.4)	8 (88.9)		
Serum Mg^2+^ (mmol/L)	0.7 (0.6, 0.7)	0.6 (0.6, 0.7)	−0.8	0.4
Serum Na^+^ (mmol/L)	137.4 ± 2.8	138.2 ± 1.7	−0.8	0.4
Serum Cl^−^ (mmol/L)	97.2 (94.4, 98.1)	94.9 (94, 98.5)	−0.7	0.5
Serum Ca^2+^ (mmol/L)	2.4 ± 0.1	2.5 ± 0.1	−1.7	0.1
Triglyceride (mmol/L)	0.9 (0.8, 1.4)	1.0 (0.8, 1.4)	−0.1	0.9
HDL-C (mmol/L)	1.3 ± 0.4	1.5 ± 0.4	−1.4	0.2
Total cholesterol (mmol/L)	4.4 ± 0.9	4.6 ± 0.8	−0.7	0.5
BUN (mmol/L)	4.1 ± 1.6	4.8 ± 1.1	−1.1	0.3
Scr (umol/L)	34.7 ± 7.9	33.9 ± 7.8	0.3	0.8
Uric acid (umol/L)	228 (187, 310)	234 (202.5, 348.75)	−0.8	0.4
Hemoglobin (g/L)	136.1 ± 6.4	135.7 ± 10.2	0.1	0.9
RASS (Supine)	21	7		
PRA [ng/(mL·h)]	2.9 (1.7, 8.6)	7.1 (2.4, 10.5)	−0.4	0.7
Angiotensin I (ug/L)	1.9 (1.3, 2.9)	2.3 (1.9, 3.3)	−0.8	0.4
Angiotensin II (ng/L)	81.8 (67.6, 91.9)	77.5 (59.5, 88.6)	−0.5	0.7
Aldosterone (ng/L)	166.5 (128.6, 202.4)	141.4 (129.5, 174.4)	−0.6	0.6
24 h Urinary Electrolytes	20	7		
Urinary K^+^ [mmol/(kg·day)]	1.7 (1.2, 2.5)	2.3 (1.7, 2.9)	−1.0	0.3
Urinary Mg^2+^ [mmol/(kg·day)]	0.1 ± 0.0	0.1 ± 0.1	−1.0	0.3
Urinary Na^+^ [mmol/(kg·day)]	5.1 ± 2.7	5.9 ± 2.9	−0.7	0.5
Urinary Cl^−^ [mmol/(kg·day)]	5.4 ± 3.1	6.3 ± 2.4	−0.7	0.5
Urinary Ca^2+^ [mmol/(kg·day)]	0.005 (0.003, 0.017)	0.006 (0.003, 0.011)	−0.4	0.7
Urinary P [mmol/(kg·day)]	0.4 (0.3, 0.6)	0.3 (0.3, 0.4)	−0.4	0.7

SD, standard deviation; HDL-C, high-density lipoprotein cholesterol; BUN, blood urea nitrogen; Scr, serum creatinine; RAAS, renin–angiotensin–aldosterone system. This table outlines the clinical phenotypes and genetic features of 30 children diagnosed with Gitelman syndrome, and further compares clinical and genetic indicators between patients stratified into functional and non-functional domain mutation subgroups. The analyzed variables encompass predominant clinical symptoms, serum biochemical data, and *SLC12A3* variant spectra. Subgroup comparison revealed no significant differences in clinical manifestations between the two cohorts. These results suggested that the classification of *SLC12A3* mutations based on functional domains cannot effectively predict disease phenotypes in pediatric GS patients.

## 4 Discussion

Gitelman syndrome (GS) is a rare hereditary renal tubular disorder. Pathogenically, pathogenic variants in the *SLC12A3* gene induce functional deficiency of the thiazide-sensitive sodium-chloride cotransporter (NCC) within the distal convoluted tubule, thereby impairing tubular reabsorption of sodium and chloride ions. Such physiological disturbance triggers compensatory activation of the renin-angiotensin-aldosterone system (RAAS), accelerates renal potassium wasting, and eventually contributes to typical GS manifestations, including hypokalemic metabolic alkalosis, hypomagnesemia, hypocalciuria, and normotension or hypotension (1, 11). In this retrospective study, we systematically enrolled 30 pediatric patients with genetically confirmed GS who were admitted to Children's Hospital of Nanjing Medical University between January 2015 and January 2025. This work aimed to expand the phenotypic spectrum of childhood GS and further explore the potential correlations between clinical characteristics and genetic profiles.

### 4.1 Clinical phenotypic heterogeneity and sex-related differences

The phenotype of GS shows significant heterogeneity in age at onset, clinical symptoms, and laboratory findings. The most common clinical symptom in this cohort was muscle weakness (50%), followed by limb numbness (40%) and short stature (36.7%). All children had hypokalemia, and 90% had hypomagnesemia at onset, consistent with the classic biochemical features of GS (1, 10). Current findings on sex differences in GS phenotype are inconsistent. In adult patients, some studies suggest sex differences: for example, a study of Chinese adult GS patients showed that males were diagnosed at an earlier age than females (13), and males may present with a more severe phenotype, including worse hypokalemia, more severe neuromuscular symptoms, and more marked urinary electrolyte excretion abnormalities. However, findings in pediatric patients are not identical. Neither this study nor a previous pediatric GS study found significant differences between male and female children in age at onset or core electrolyte indices such as serum potassium and magnesium (3, 22). Notably, this study found that the incidence of limb numbness was significantly higher in female children, with relatively lower serum calcium levels. Previous studies have indicated that female patients with GS may be more sensitive to electrolyte disturbances, particularly abnormalities in calcium and magnesium metabolism (24, 25). Furthermore, total symptom scores in adult female GS patients have been shown to be significantly higher than in males, suggesting a potentially higher symptom burden or a greater propensity to express symptoms in female patients (26). Taken together, our findings suggest that in pediatric patients with GS, gender differences may primarily manifest in the frequency and subjective perception of neuromuscular symptoms, rather than in the severity of core electrolyte disturbances. In clinical practice, it is crucial to pay close attention to the neuromuscular manifestations in female patients, comprehensively assess their calcium and magnesium metabolic status, and implement individualized management strategies.

### 4.2 Severity of hypokalemia: association with clinical symptoms and metabolic disorder risk

In this study, children were grouped by serum potassium level. The severe hypokalemia group (K^+^<2.5 mmol/L) had a significantly higher incidence of tetany than the mild-to-moderate group, accompanied by lower serum sodium levels. This finding is consistent with the core pathophysiology of GS: inactivation of NCC causes sodium and chloride loss (salt wasting), relative hypovolemia activates RAAS, which promotes sodium reabsorption while exacerbating potassium excretion and metabolic alkalosis (1, 13, 27). Severe hypokalemia is usually associated with more marked sodium loss and RAAS activation (28). Notably, this study found that children in the severe hypokalemia group already showed a trend of dyslipidemia with elevated triglycerides (TG) and decreased high-density lipoprotein cholesterol (HDL-C). This finding suggests a potential association between childhood electrolyte disturbances and the risk of long-term metabolic complications. The potential mechanism may involve chronic hypokalemia and hypomagnesemia, which are known triggers of insulin resistance. Chronic hypokalemia blocks the closure of ATP-sensitive potassium channels on pancreatic β-cells, reducing insulin secretion, while hypomagnesemia decreases insulin receptor tyrosine kinase activity and peripheral tissue insulin sensitivity (6). Insulin resistance and compensatory hyperinsulinemia are the core pathological basis of dyslipidemia (high TG, low HDL-C) (6). Numerous studies confirm that adult GS patients are at high risk of type 2 diabetes. A study of 67 Chinese GS patients showed that 19.4% had type 2 diabetes and 20.9% had impaired glucose tolerance, with elevated insulin resistance indices (13). Another study also noted that GS patients with severe hypomagnesemia had more marked insulin resistance (6). Therefore, severe hypokalemia and early dyslipidemia in childhood may indicate an increased risk of subsequent overt glucose metabolism disorders. This suggests that clinical management for these children should not be limited to correcting electrolytes; blood glucose, insulin, and lipid levels should be monitored early and long-term, and active, continuous potassium and magnesium supplementation may help improve insulin sensitivity and prevent or delay long-term metabolic complications such as diabetes (6, 13).

### 4.3 Genotype–phenotype correlation analysis: limitations of functional domain classification

The genotype-phenotype relationship in Gitelman syndrome (GS) is complex and not yet fully elucidated. Previous studies have employed various classification schemes (e.g., based on mutation type, zygosity, or predicted functional impact) for correlation analysis. While some differences in specific indicators have been found, the results have often been inconsistent (13, 19, 22). In this study, we grouped and compared patients based on whether SLC12A3 variants were located in known functional domains of the protein. Similarly, we found no statistically significant differences between the two groups in major clinical and laboratory indices. This negative finding reflects the well-recognized complexity of the genotype-phenotype relationship in GS, which stems from multiple factors: (1) Diverse pathogenic mechanisms of mutations: SLC12A3 exhibits a wide variety of mutations, with missense mutations being the most common (17, 26, 29). The mechanisms by which mutations affect NCC function are complex and not solely determined by their location in classic functional domains. For example, the common Chinese mutation p.Asp486Asn (D486N), located in the intracellular loop, causes disease primarily by reducing total and membrane NCC expression and impairing its membrane localization. Many missense mutations may cause protein misfolding, retention, and degradation in the endoplasmic reticulum, or impair trafficking to the apical membrane—mechanisms that cannot be predicted by simple “functional domain” localization (1, 27). (2) Genetic heterogeneity and modifier factors: A considerable proportion (8%–40% reported in the literature) of clinically diagnosed GS patients carry only a single heterozygous SLC12A3 mutation (3, 4, 30), suggesting the presence of deep intronic mutations, large deletions/duplications not captured by conventional WES, or modifier effects of other genes (e.g., CLCNKB, HNF1B) that collectively influence the final phenotype (17, 27, 29). In addition, differing mutation hotspot distributions across ethnic groups may also confound correlation analyses (19, 29). Given these complexities and the limitations of the current study, future prospective, multi-center, large-sample studies combined with *in vitro* functional assays (e.g., Western blot, immunofluorescence for protein expression and localization) to verify the molecular mechanisms of specific mutations will help to more accurately elucidate the genotype-phenotype association.

### 4.4 Limitations of the study

Despite several valuable findings, this study has multiple limitations. First, the small sample size of 30 patients limited result generalizability and statistical power, especially in subgroup analyses. Considering the small cohort, multiple comparison correction was not performed to avoid reduced sensitivity and false-negative results. However, multiple statistical tests may increase false-positive risks; thus, all significant findings should be interpreted cautiously. This issue is common in rare disease research, including GS. Second, the retrospective design carried potential selection and information bias. Data retrieved from medical records may contain missing information and inconsistent documentation, which could be improved via prospective data collection. Third, genotype–phenotype analyses relied solely on bioinformatic predictions without functional validation. *In vitro* experiments are required to confirm variant pathogenicity, and current correlations remain speculative.

Finally, this single-center study could not fully represent pediatric GS patients across China. Large-scale, multicenter studies are needed to validate our results and resolve the above limitations.

## 5 Conclusion

This study, by analyzing 30 pediatric GS patients, further delineates the clinical and genetic characteristics of this disease in the Chinese population. We found that female children had more prominent limb numbness and relatively lower serum calcium levels, suggesting that sex may influence symptom expression. Severe hypokalemia may not only serve as a marker of disease severity; its associated early dyslipidemia may indicate an increased risk of long-term insulin resistance and diabetes, suggesting the need for close clinical attention and early intervention. Furthermore, functional domain classification based on bioinformatic prediction failed to effectively predict phenotype, reflecting the complexity of the GS genotype–phenotype relationship, and highlighting the need for further exploration combined with functional studies in the future.

## Data Availability

The data analyzed in this study is subject to the following licenses/restrictions: The dataset analyzed in this study is derived from retrospective routine clinical data collected for diagnostic and therapeutic purposes. Due to ethical restrictions and patient privacy protection, the raw clinical and genetic data cannot be made publicly available. Access to the dataset may be granted only upon reasonable request and with approval from the corresponding authors and the institutional ethics committee. Requests to access these datasets should be directed to Xueqin Cheng, cxq80416@163.com.
